# Validation of an Inhaled Therapy Beliefs Questionnaire in Patients with Chronic Obstructive Pulmonary Disease

**DOI:** 10.3390/jcm13082281

**Published:** 2024-04-15

**Authors:** Francisca Muñoz-Cobos, Virginia P. Aguiar-Leiva, Carmen Argüello-Suárez, Paula Colacicchi, Luis Antonio Calleja-Cartón, Francisca Leiva-Fernández

**Affiliations:** 1 Andalusian Health Service, Málaga Biomedical Research Institute (IBIMA-Plataforma BIONAND), University of Málaga, 29590 Málaga, Spain; 2 Research Unit Instituto CUDECA de Estudios e Investigación en Cuidados Paliativos Fundación CUDECA, Biomedical Research Institute (IBIMA-Plataforma BIONAND), University of Málaga, 29590 Málaga, Spain; 3 Multiprofessional Teaching Unit for Family and Community Care of the Málaga-Guadalhorce Primary Care District, 29009 Málaga, Spain; 4 Andalusian Health Service, 29010 Malaga, Spain

**Keywords:** COPD, chronic diseases, primary care, medication adherence, patient medication knowledge, nebulizers and vaporizers

## Abstract

**Background**: To carry out a validation questionnaire that assesses beliefs about inhaled treatments in patients with chronic obstructive pulmonary disease (COPD), as knowing patients’ beliefs could help to improve medication adherence and health outcomes. **Methods**: We evaluated data from 260 COPD patients from electronic medical record databases from five primary healthcare centers, in a descriptive, cross-sectional study with a sample size calculated for a 10-item questionnaire, with an estimated Cronbach’s alpha of 0.70 and a 95% confidence level. Study participants were selected via systematic random sampling. Variables: Ten-item Inhaled Therapy Beliefs Questionnaire, CCTI-Questionnaire v.2.0, time for completion, age, sex, educational level, spirometry severity (GOLD criteria), exacerbations (previous year), characteristics of inhaled treatment, and smoking habit. A two-year follow-up in a subsample of 77 patients from one health center was utilized. The Morisky–Green test, pharmacy dispensing data, test–retest (kappa coefficient), and an exploratory analysis of the adherence–belief relationship (ji-squared) were measured. **Results**: The 10-item questionnaire showed good viability (3 min completion time) when performed face-to-face or telephonically; its psychometric properties were acceptable, with an internal consistency (Cronbach’s alpha) score of 0.613. Three factors explained 47.58% of the total variance (*p* < 0.0001): use (factor 1), effects (factor 2), and objectives (factor 3) of inhalers. The two-year follow-up ultimately considered 58 out of the 77 patients (10 deceased, 4 unlocated, 2 mistakes, 2 no inhaled treatment, and 1 withdrawal). Non-adherence was 48.3% in terms of the Morisky–Green test; 31% in terms of pharmacy dispensing data; and 40.4% considering both methods. There was low test–retest reliability, indicated by items 4, 8, and 9 of the CCTI-Questionnaire (Kappa = 0.4, 0.26, and 0.34; *p*-value < 0.0001, 0.008, and 0.001, respectively). There was mild correlation between beliefs and adherence. **Conclusions**: The ten-item CCTI-Questionnaire v.2.0 demonstrated acceptable psychometric properties regarding feasibility, reliability, and content validity.

## 1. Introduction

Chronic obstructive pulmonary disease (COPD) is a highly prevalent chronic disease. According to the EPI-SCAN II study, a national multicentric cross-sectional population-based epidemiological study, it affects 11.8% of the population over 40 years of age in Spain, with significant differences in prevalence between men (14.6%) and women (9.4%). Prevalence increases with age, peaking after 80 years at 34.7% in men and 26.1% in women [1].

The beneficial effects of inhaled treatments include improved lung function, reduced exacerbations, decreased dyspnea, and improved quality of life [1]; however, poor adherence to this treatment has been described. The reasons for poor adherence include treatment characteristics, such as difficulties using a device, the level of instruction received [2], and the type and diversity of devices [3,4], as well as complex regimens, adverse effects, and perceptions about treatment efficacy. Other patient-related factors that affect treatment adherence include socioeconomic level [5], cultural factors and beliefs, cognitive status [6], and the burden of the treatment [7].

The relationship between health beliefs and behaviors was highlighted by the Health Belief Model developed in the 1950s by the American Public Health Service. Belief is a subjective truth, a conviction or something that a subject holds to be true, and is not to be confused with objective truth, as the subject does not relate to reality but to one’s (mental) representation of it. The Health Belief Model intended to explain the lack of public participation in early disease detection and prevention programs. Subsequently, the model was adapted to better understand a variety of health behaviors, including an individual’s response to certain symptoms of illness and patient compliance with treatments as well as medical recommendations [8].

Beliefs about inhaled treatments are part of a patient’s mental representation of COPD. Unlike some chronic diseases, such as cancer [9], AIDS [10], and diabetes [11], for which there are well-defined individual and social mental models, COPD is a rather confusing entity for affected patients [12,13].

In the case of COPD patients, it has been found that health behaviors tend to be consistent with the mental construct of disease, with the perception of the ability to intervene and influence the course of the disease (self-efficacy) being very relevant [13,14].

Patients’ experiences, beliefs, and mental models shape their behaviors towards their illnesses and are considered indicators of quality of care and prevention practices, given their relationships with health outcomes [15,16]. The patient experience is a multidimensional construct associated with multiple domains, which include a patient’s physical and emotional life experience, their interactions with healthcare providers, the culture of the healthcare organization, the level of patient involvement, continuity of care, and patient-centered care [17,18]. Other authors have included patient experience as an evaluative element of comprehensive care in the context of physical and mental comorbidity [19].

Vogelmeir et al. [1] have studied the impact of patients’ beliefs on treatment adherence in patients with chronic diseases, including COPD, finding statistical significance in this association in 80% of the included studies, although the direction of the belief–adherence relationship varies according to the factors analyzed (especially religious beliefs, control beliefs, and knowledge of the disease), leaving a gap in the research on this issue [20].

Horne et al. [21] have studied the influence of disease representation on treatment adherence in patients with chronic diseases, showing a high correlation between the “need/concerns framework” construct for grouping patients’ beliefs (perceived need for medication/concerns about dependence and long-term adverse effects) and treatment adherence. They concluded that medication beliefs were more powerful predictors of reported adherence than clinical and sociodemographic factors, accounting for 19% of the explained variance in adherence.

Horne et al. [22] have delved into the application of this model, which has reported that adherent patients have a strong perception of a need for treatment and that this relationship is significant across countries, sample sizes, and methods of measuring adherence [22]. This perception of the need for treatment varies according to the intentionality of non-adherence: patients with intentional non-adherence had a lower perception of the need for their medication and a higher level of concerns about it due to adverse effects or habituation, among other aspects, compared to adherent patients [23]. Recent research confirms that the perceived need for inhaled medication is related to increased adherence, while the belief that these medications are harmful is a determinant of poor adherence [24].

The influence of beliefs on adherence has also been measured by qualitative studies, finding different perceptions and beliefs in patients with inhaler overuse or underuse [25].

Thus, it is crucial to assess COPD patients’ beliefs about their treatments. Questionnaires are the instruments most widely used to assess patients’ beliefs. Recently, specific questionnaires for patients with COPD include the COPD-Patient Outcome Report [26], which contains a single item on the perception of treatments (“satisfaction with the effect of treatment”); the test of adherence to inhalers, which includes two items on beliefs about inhalers, although the objective is to evaluate adherence [27]; and the Disease Awareness in COPD Questionnaire [28], which includes nine statements on inhaled treatments out of the twenty that make up the questionnaire, and broadly evaluates disease knowledge, perception, and acceptance. All three questionnaires include some aspects related to beliefs about inhaled treatments but are designed with more global objectives.

Therefore, a specific questionnaire on beliefs about inhaled therapy has been designed for use in clinical practice. The aim of this article is to validate the Inhaled Therapy Beliefs Questionnaire, CCTI questionnaire (from its acronym in Spanish) version 2.0, in patients with COPD, with prescriptions of continuous inhaled treatments.

## 2. Materials and Methods

**Figure 1:**
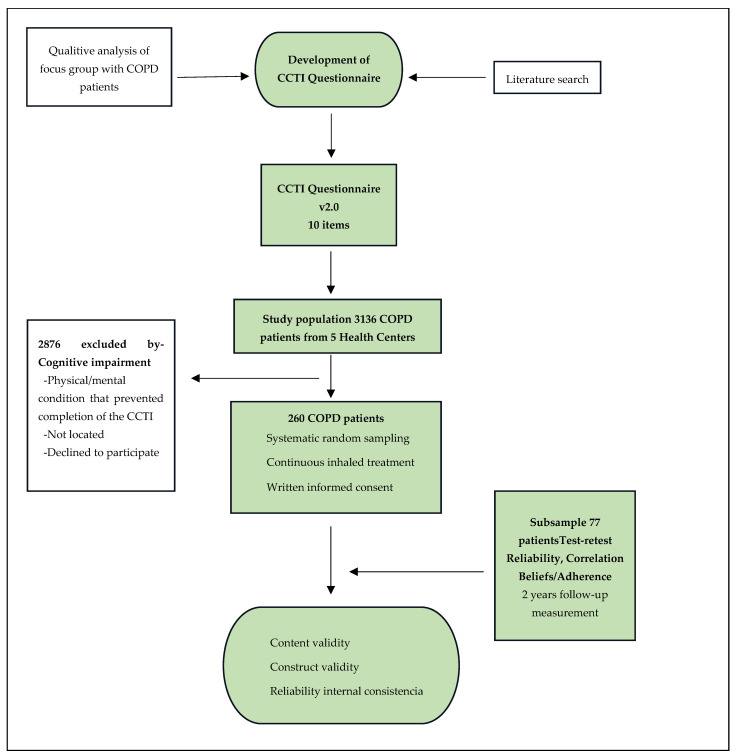
Flow diagram of the study. COPD: chronic obstructive pulmonary disease; CCTI: Inhaled Therapy Beliefs Questionnaire (Spanish acronym); and v.2.0: version 2.0.

The validation study was carried out in 5 health centers in Malaga: 4 in urban areas and 1 in a rural area. A descriptive, cross-sectional study design was used, and the flow diagram of the study is represented in Figure 1.

The study population consisted of COPD patients who were registered in the electronic medical record databases (as of August 2018) of the 5 participating centers (N = 3136) and had prescriptions for continuous inhaled treatments. All participants provided written informed consent. Patients with a cognitive impairment or a physical/mental condition that prevented the completion of the questionnaire were excluded.

The final sample comprised 260 patients. The sample size was calculated for a 10-item questionnaire for an estimated Cronbach’s alpha of 0.70, with a 95% confidence level, an effect size of 1.1, a statistical power of 80%, and a predicted study dropout rate of 30%. Study participants were selected via systematic random sampling.

The selected patients were contacted by telephone and offered the opportunity to participate in the study. If they failed to meet the inclusion criteria, could not be located, or declined to participate, the next patient on the sampling frame was selected.

The primary outcome variable was beliefs about inhaled treatments measured using the Inhaled Therapy Beliefs Questionnaire, CCTI questionnaire version 2.0 (Spanish acronym).

The steps for the development of the CCTI questionnaire have been previously published [29]. To summarize, a list of inhaler beliefs from a qualitative study with video-recorded focus group interviews of COPD patients was selected for the questionnaire [13]. Bibliographic contributions were added [30], resulting in 20 judgements that could be assessed as true/false. The researchers agreed on the relationship of each item to the belief explored. Face validity was assessed by 10 professionals who routinely care for COPD patients (family medicine and nursing from a primary care setting, pulmonology, emergency from a hospital setting, and office pharmacy) using convenience sampling. With the contributions of these professionals, the initial questionnaire was modified.

To assess feasibility, a two-phase pilot study was designed. The version modified by expert opinion was presented in a self-administered form to 23 randomly selected patients who were invited to a group session. The aim was to assess feasibility by measuring comprehension, acceptability, and completion time. The new version of the questionnaire was piloted individually under real clinical practice conditions. Patients from urban and rural health centers were included. For this second piloting, 26 subjects were selected via consecutive purposive sampling in family medicine consultations during February 2019. The research team carried out a final selection of items based on the following criteria: (1) confusion: existence of unclear aspects in the statement subject to different interpretations by the patient (relevant percentage of “no answer”); (2) redundancy: there were other sentences exploring the same belief; (3) lack of comprehension: the wording was not understood; and (4) degree of applicability to all COPD patients (only to those using certain inhaler devices). Finally, version 2.0 of the CCTI questionnaire was drafted consisting of 10 items [29], with a true/false/no answer format, the results of which were expressed as the percentage of correct answers/incorrect answers/non-answers.

For each item, it was established what type of response should be considered correct (correctly marking a statement as true or false) or incorrect (marking a true statement as false or vice versa). This was carried out based on a review of the literature, after establishing consensus among the research team, and following a review performed by the panel of experts that participated in the pilot study [29]. The global numerical score consisted of the sum of the total number of correct responses. This version of the questionnaire (version 2.0) has been validated in the present study.

Data collection for validation was initially carried out through individual interviews. Participating patients were contacted by telephone (up to 3 calls) and informed of the day, time, and place (their reference healthcare center) at which the individual interview would take place. Once written informed consent was provided, study variables were collected and the patient was presented with the questionnaire for completion, along with instructions on how to do so and answers to any doubts they had. All doubts were noted by the researcher, as well as the time taken to complete the questionnaire. Subsequently, with the onset of the COVID pandemic, interviews were conducted by telephone after a new ethics committee approval.

The following independent variables were used: age, sex, educational level, rural/urban environment, and spirometry severity (Global Initiative for Chronic Obstructive Lung Disease—GOLD—criteria 2022) [31] from a patient’s electronic clinical record, the number of exacerbations in the previous year, years since disease onset, duration of inhaler use, type of inhaled treatment (pharmacological group, type of device, prescribed daily dose, number of different inhaled drugs, and number of different devices), current smoking status, and number of pack-years. We did not include the presence of vapers or dual cigarette–vapers among the study participants.

The time taken to complete the questionnaire and the modality used (face-to-face or telephone) were also recorded.

Test–retest reliability and sensitivity to change were evaluated in a subsample of 77 patients from one of the five health centers of the study, two years after the first application. In this group, the persistence to the inhaled treatment prescribed on a continuous basis was considered according to the Ascertaining Barriers to Compliance (ABC) taxonomy [32] and was assessed using the Morisky–Green test in its version for inhaled treatments [33]. The dispensing rate, as another measurement of medication adherence, was calculated considering the ratio between the number of canisters withdrawn at the pharmacy with respect to the theoretical number that should be used according to the prescription schedule. The construct validity of the CCTI questionnaire was assessed by considering the relationship between the measures of medication adherence and the beliefs assessed by the CCTI questionnaire, testing the hypothesis that correct beliefs will be related to better adherence to medication.

### Statistical Analysis

The descriptive analysis of all study variables was carried out by calculating percentages for qualitative variables and the mean, median, and standard deviation for quantitative variables. The validity methods used were a reliability analysis, to assess the internal consistency of the questionnaire, and a factor analysis to test the construct validity. To evaluate internal consistency, we used Cronbach’s alpha: a value > 0.7 was considered acceptable [34]. In addition, we assessed the correlation between each element and the global numerical score and determined Cronbach’s alpha after individually eliminating each element. An exploratory factor analysis was conducted to examine the underlying or latent dimensions or constructs of the variables [35]. The principal component technique was applied using a varimax rotation, and its applicability was assessed using the Kaiser–Meyer–Olkin (KMO) test and Bartlett’s sphericity test (considering a *p*-value < 0.05 as ideal). Cronbach’s alpha was then calculated separately for each of the factors identified in the factor analysis. An analysis of the retest reliability using the Kappa coefficient of concordance was applied, considering acceptable values higher than 0.40 [36]. A comparison of before–after patient characteristics was conducted via the McNemar test and paired-sample *t*-test as appropriate to the nature of the variables being compared.

The study was approved by the Research Ethics Committee of the Province of Malaga on 3 July 2019 and again on 7 January 2020 after modification of the data collection method due to the COVID pandemic.

## 3. Results

The final study population consisted of 262 patients (Figure 1). The distribution of participants according to healthcare center and the modality of administration of the questionnaire are shown in Table 1.

We only had access to a rural center and, due to its under-representation, an analysis of the difference between urban and rural areas was not conducted.

The sociodemographic and clinical characteristics of the participants are shown in Table 2: 75% were men (198 out of 262), the median age was 72 years (minimum 47 years, maximum 92 years), 80,9% (212 of 262) had a medium-to-low educational level, 74% (193 of 261) were ex-smokers, and 70,2% (184 out of 262) had mild-to-moderate COPD severity.

We analyzed the differences in the sample regarding the main health outcomes and treatments prescribed.

The responses to the questionnaire are shown in Table 3. The mean (SD) global score was 6.43 (2) out of a maximum of 10 points (median: 7 points). A score of 5 or more was obtained by 69.5% of patients. The mean (SD) completion time was 3.11 (1.53) minutes (median: 3 min).

For validation statistics, questionnaire responses classified by the researchers as correct or incorrect were used. The internal consistency of the questionnaire was assessed using Cronbach’s alpha, for which a score of 0.613 was obtained.

The item–total correlation is presented in Table 4. For all items we observed correlations > 0.2, except for the following questions: “Inhalers are used to open the bronchial tubes and let in more air” (ICC, 0.187) and “If I use inhalers daily, breathlessness will decrease” (ICC, 0.155).

The suitability of performing the factor analysis was verified in the 10-item version of the questionnaire (Kaiser–Meyer–Olkin test, 0.74; Bartlett’s sphericity test, *p* < 0.0001) and three factors were identified that explained 47.58% of the total variance. Factor 1, relating to the use of inhalers (items 4, 5, 7, 8, and 9 of the questionnaire), explained 21.76% of the variance; factor 2, relating to the effect of inhalers (items 2, 3, and 6 in the questionnaire), explained 13.18% of the variance; and factor 3, relating to the objective of the inhaled treatment (items 1 and 10), explained 12.64% of the variance (Table 5).

The internal consistency of these three factors was separately assessed using Cronbach’s alpha, with different results among factors: factor 1 had a Cronbach’s alpha of 0.74; factor 2 had a Cronbach’s alpha of 0.15; and factor 3 had a Cronbach’s alpha of 0.38.

The two-year retest was analyzed in the subgroup of one of the selected health centers, including 58 of the 77 patients from the initial sample participating in the first completion of the CCTI. Losses were due to the following causes: 10 deceased (12.9%), 4 unlocated (5.1%), 2 included by mistake (2.5%), 2 no longer prescribed inhalers (2.5%), and 1 voluntary withdrawal (1.2%). Their characteristics were similar to the overall sample except for the following aspects: spirometry severity (44.4% of initial severe COPD relapsed to moderate and 1% to mild, *p* < 0.0001) and smoking (6.7% of former smokers relapsed and 25% of initially active smokers stopped smoking, *p* < 0.0001). Regarding treatments, the number of different devices used decreased from 1.76 (SD: 0.57) to 1.57 (SD: 0.67) (*p* = 0.055); the use of Respimat (34.5% vs. 17.2% at the baseline) became more important, and the use of Handihaler (22.4% at the baseline to 1.7% at the second assessment) was less frequent. The type of drug also showed differences: the combination of long-acting bronchodilator inhalers (LABA) + corticoid went from 34.5% to 27.6% (*p* < 0.0001), long-acting muscarinic receptor antagonists (LAMA) went from 36.2% to 24.13% (*p* < 0.0001), and the triple combination of LAMA + LABA + corticoid went from 6.9% to 10.7% (*p* < 0.005).

In this subgroup, adherence (assessed as persistence according to the ABC Taxonomy [32]) in the second determination of the CCTI questionnaire version 2.0 according to the Morisky–Green test yielded the following results: 48.3% were non-adherent. According to pharmacy dispensing data, 31% of patients were non-adherent. When analyzed with both methods, 40.4% of patients were non-adherent.

The concordance analysis showed a low test–retest reliability, with only weak concordance found in some questions (items 4, 8, and 9 of the CCTI questionnaire) (Kappa coefficient = 0.4, 0.26, and 0.34; *p* < 0.0001, 0.008, and 0.001, respectively).

A relationship was found between adherence and the belief of abandoning treatment if improvement occurs (“*When I get better, I have to stop using the inhaler*”), applying to 74% of adherent patients and only 47% of non-adherent patients (*p* < 0.002).

## 4. Discussion

Based on our findings, we present a 10-item questionnaire for the assessment of COPD patients’ beliefs about inhaled treatments (the CCTI questionnaire version 2.0) that shows good viability, with a mean completion time of 3 min, whether performed face-to-face or telephonically.

The questionnaire’s psychometric properties are acceptable, but the global Cronbach’s alpha value is lower than expected (0.613), although it is close to the established limit of 0.7. The reliability of an instrument is closely associated with its validity, in such a way that an instrument cannot be valid unless it is reliable. There are multiple definitions and types of reliability measurements; one of the most commonly used is Cronbach’s alpha [37]. The most accepted values of Cronbach’s alpha range from 0.70 to 0.95. A low value of Cronbach’s alpha could be due to a low number of questions, poor inter-relatedness between items, or heterogeneous construct validity [38]. In our case, as the CCTI questionnaire version 2.0 is short (10 items), this could have played a role in the lower Cronbach’s alpha value. The results obtained when analyzing the Cronbach’s alpha in each of the subscales of the questionnaire (factor 1, factor 2, and factor 3), following the recommendations of some authors [39], lead us to believe that the internal structure of the questionnaire pivots mainly on Factor 1, whose items reflect the same construct, stemming on a unidimensional scale, thus providing a more “interpretable” result.

The homogeneity of the scale assessed using the item–total correlation was adequate (with values > 0.20, except for items 6 and 10). In the case of item 6, the item–total correlation was at the limit (0.18). This may be due to the non-discriminatory nature of item 6 (*Inhalers are used to open the bronchial tubes and let in more air*). Indeed, the percentage of correct answers was over 90%, perhaps due to the general definition of the bronchodilator effect of inhalers, and also because this statement is a demonstrably correct belief held by the patients who participated in the study, who all had long periods since disease onset and long durations of inhaler treatment. A high percentage of correct answers was also observed for item 10 (*If I use inhalers daily, I will suffocate less*). The individual elimination of either of these items did not improve Cronbach’s alpha. These findings could be due to a ceiling effect in both situations. Ceiling and floor effects indicate that a measure is missing important information [40], and there is controversy as to whether it is appropriate to consider these values true or eliminate them [41]. Finally, it was decided to maintain these two items in the questionnaire, considering that they could serve as an educational reinforcement for the patients to whom the tool was applied, exploring the persistence of this ceiling effect in future studies.

The factors identified correspond to the four main variables in the Health Belief Model. The original version of the Health Belief Model [42] describes four key variables that appear to influence a subject when performing a given health behavior. These variables are grouped around two main dimensions: (i) the degree of willingness of a subject to carry out an action, which is determined by perceived vulnerability and severity; (ii) the perceived costs and benefits associated with the action to be performed.

The combination of the first two variables (perceived vulnerability and severity), which reflect the degree of the “threat” posed by the disease, determines a subject’s motivation to act. The specific behavior that a subject adopts will be that which reduces the threat of the disease with the greatest perceived benefit and the lowest perceived cost.

The evaluation performed by an individual before they carry out a health behavior (in our case: treatment adherence persistence) is made up of four dimensions: (i) *perceived susceptibility (vulnerability)*: the subjective assessment of the possibility of suffering the effects of the disease (symptoms, functional interference, and impact on quality of life) if treatment is not carried out; (ii) *perceived severity*: the subjective perception of the seriousness and risks posed by the untreated disease (decompensation, hospitalization, and death); (iii) *perceived benefits*: the perceived benefits of undergoing treatment, the perception of the degree to which the recommended actions are effective in treating/managing the disease; and (iv) *perceived barriers (costs)*: the difficulties, potentially negative aspects, adverse effects, and/or obstacles to following the treatment recommendations and aspects that can make the treatment difficult or burdensome. These factors should be considered as a counterweight to the aforementioned dimensions.

In the CCTI questionnaire, factor 1 encompassed beliefs relating to the use of inhalers. This factor includes items 4, 5, 7, 8, and 9. These items reflect beliefs that lead to direct decisions as to whether or not to use an inhaler on a daily basis. They correspond to the question “*Should I use the inhaler today/now?*”. There is a direct and daily relationship between these beliefs and the action. It involves an assessment of the dimensions of the Health Belief Model: susceptibility–severity–barriers, which counterbalance the benefits represented by factor 2. Each person, regardless of their prescribed regimen, has a pre-formed personal pattern of inhaler use that influences their decision as to whether or not to administer a dose. This is predetermined, automatic, and routinized: it occurs unconsciously. Patients do not make a decision every time they administer a dose, unless certain circumstances have changed. It is determined by personal experience and beliefs, and can change, albeit slowly. It is concordant with the high frequency of non-deliberate non-adherence in patients with COPD [43].

Factor 2 encompasses beliefs about the effects of inhalers. This factor includes items 2, 3, and 6. These items reflect the expectations of a prospective user about the effects (or benefits, according to the Health Belief Model) of inhaled treatments. This is less closely related to the decision to use an inhaler, and corresponds more to the reasoning or justification behind that decision.

Similar results have been reported in qualitative studies that demonstrate the importance of sensory markers of inhalation and of the expectation of a fluidizing effect for compliance with inhaled treatments [13].

Factor 3 encompasses beliefs about the goals of treatments. This factor includes items 1 and 10. These lie on a mental plane and are further removed from the more practical aspects of inhaler use, and correspond to the reasons provided to a patient by a health professional to “convince” them of the need for inhaled treatments. Both items reflect a generic benefit according to the Health Belief Model.

The low reliability of responses at two years may be influenced by the length of this period and the changes that have occurred during this time, in patients and their treatment, as well as the changing nature of beliefs and the multiple factors that affect them. This long period of time allows the recall effect of previous responses to be ruled out. The reduction in the number of devices and changes in treatments may reflect the influence on prescribing of updated COPD treatment recommendations.

Rates of non-adherence to inhaled therapy are similar to those reported in the literature and vary according to measurement methods, but are always high, reaching 50%, with some studies [41] exceeding this figure and characterizing non-adherents as younger and black or Hispanic, with a lower economic status and fewer years of formal education, as well as having more severe COPD.

With regard to the results of the analysis of the relationship between beliefs and adherence, considered in the phase of persistence with the prescribed treatment, the sample would have to be larger than the one recruited to improve the ability to establish this relationship; initially, it seems that the item most closely related is the one referring to stopping the use of an inhaler when the clinical situation improves.

The perceived need for treatment appears as a belief strongly related to adherence to inhaled therapy in patients with chronic respiratory diseases (asthma and COPD) [44]. A study using a qualitative and quantitative methodology shows that the beliefs about medicines questionnaires’ (BMQ) need scale score was significantly higher in adherent patients (*p* = 0.000) [45]. In the qualitative part of this study, using semi-structured interviews, some of the reasons for poor adherence included a fear of side effects, concerns about toxicity and long-term tolerance (consistent with the cited aspect of concern about treatments in Horne’s model [21]), discontinuing medications if there were fewer symptoms, and use only if necessary. Other studies have shown the predictive character of patients’ concerns about medications and adherence to treatments [46].

The beliefs about continued inhaler use being harmful (better not to use daily, use as little as possible) are relevant to our questionnaire, in reference to the Horne model’s “concerns about treatment” factor. These beliefs are included in factor 1 and are coincident with the BMQ’s “Belief that medicines are harmful” factor, which is related to low adherence [24].

In a systematic review conducted in 2019 focused on patients with several chronic conditions (diabetes, hypertension, asthma, and COPD) a significant positive or negative association between personal as well as cultural beliefs and medication adherence was highlighted. The authors pointed out that it is necessary to explore beliefs systemically and work towards changing those that are negative with regard to the perception of the disease and the different options for its management, including drug treatments [20].

This study has several limitations: The main concern regarding the reliability of the study is the long time elapsed between the first and the second measurements. This may have introduced various confounding factors that could have affected both the beliefs and behaviors of the study subjects. Therefore, these results should be taken with caution. We plan to replicate this study by assessing patients’ beliefs with this questionnaire at shorter time intervals and in larger numbers, in order to show the evolution and relationship between beliefs and behaviors. Second, due to the pandemic, we had to change the procedure for administering the questionnaire (from face-to-face to over the telephone). Third, there is a possibility that recall bias may have affected the recording of certain variables during the interviews. Fourth, some variables were taken from patients’ clinical records and there may have been some errors in data recording, which may have resulted in the loss of some information.

As for its main strengths, we consider the elaboration of the first questionnaire completely aimed at assessing beliefs in inhaled therapy in this study population, following a rigorous procedure and with the participation of the patients themselves in the construction of its items. Another important characteristic is its briefness, with a sufficiently short application time requirement to make it usable in any healthcare setting (primary care or hospital environment).

Further studies will be required to verify the functioning of the questionnaire in clinical practice and its psychometric properties in large and distinct groups of patients. The detection of erroneous beliefs will facilitate the design of strategies to correct them, thus promoting better adherence to inhaled treatments and better disease control.

## 5. Conclusions

In conclusion, the findings presented here support the acceptable psychometric properties (regarding feasibility, reliability, and content validity) of a 10-item Inhaled Therapy Beliefs Questionnaire (the CCTI questionnaire version 2.0), to identify beliefs about inhaled treatments in COPD patients, as a first step to gaining a closer understanding of patients’ perspectives on their treatments, to be able to implement actions aimed at correcting false beliefs, and consequently achieve better adherence as well as better health outcomes derived from the better use of treatments that have been shown to be effective for COPD.

## Figures and Tables

**Table 1:** Distribution of patients included by health centers and interview modality.

Health Center	Environment	N	n	Total Included n = (n_p_ + n_t_)	Physical Interview n_p_ (%)	Telephone Interview n_t_ (%)	Percentage of the Total Sample
El Palo	Urban	925	76	77	53 (69)	24 (31)	29.4
Alameda-Perchel	Urban	634	53	53	11 (21)	42 (79)	20.2
Rincón de la Victoria	Urban	936	77	78	9 (12)	69 (88)	29.8
Puerto de la Torre	Urban	532	44	44	12 (27)	32 (73)	16.8
Colmenar	Rural	109	10	10	7 (70)	3 (30)	3.8
		3136	260	262	92 (35.1)	156 (64.9)	100

N = total number of patients with chronic obstructive pulmonary disease in each health center; n = sample size; n_p_ = number of patients assessed via a physical interview; and n_t_ = number of patients assessed via a telephone interview.

**Table 2:** Sociodemographic and clinical characteristics of the participants.

Variable	Result
Age: mean (SD), median, years	71.5 (8.1), 72
Gender: male, n (%)	198 (75.6)
Urban environment: n (%)	249 (95)
Education level: n (%)	
Lower to primary level	19 (7.3)
Primary level	124 (47.3)
Secondary level	69 (26.3)
Higher education	43 (16.4)
Missing values	7 (2.7)
Smoking habit: n (%)	
Never	7 (2.7)
Ex-smoker	193 (73.9)
Active smoker	55 (21.1)
Passive smoker	6 (2.3)
Tobacco use packs/year: mean (SD), median	48.4 (31.6), 41
Severity ^a^ n (%)	
Mild	51 (19.5)
Moderate	133 (50.8)
Severe	64 (24.4)
Very severe	7 (2.7)
Missing values	7 (2.7)
Exacerbations last year: mean (SD), median	0.6 (1.05), 0.0
COPD evolution time (years since diagnosis): mean (SD), median	8.9 (5.11), 8.0
Time with inhalers: n (%)	
Less 1 year	7 (2.7)
Between 1 and 5 years	86 (32.8)
Between 6 and 10 years	72 (27.5)
More than 10 years	96 (36.6)
Missing Values	1 (0.4)
Inhaled drug used: n (%)	
SABA	70 (26.7)
SAMA	51 (19.5)
LABA	16 (6.1)
LAMA	103 (39.3)
CORTICOIDE	13 (5.0)
LAMA + LABA	91 (34.7)
LABA + CORTICOID	97 (37.0)
LAMA + LABA + CORTICOID	19 (7.3)
SABA + CORTICOID	1 (0.4)
Number of inhaled drugs: mean (SD), median	1.79 (0.73), 2.0
Number of inhalation devices: mean (SD), median	1.73 (0.67), 2.0
Type of inhalation devices: n (%)	
Pressurized metered-dose inhalers	95 (36.6)
Turbuhaler	84 (32.1)
Handihaler	61 (23.3)
Breezhaler	61 (23.3)
Novolizer	46 (17.6)
Respimat	39 (14.9)
Accuhaler	14 (5.3)
Genuhair	12 (4.6)
Spiromax	8 (3.1)
Nexthaler	6 (2.3)
Easyhaler	4 (1.5)
Zonda	3 (1.1)
Twisthaler	1 (0.4)
Aerolizer	1 (0.4)

SD: standard deviation; ^a^ severity according to GOLD 2019; SABA: short-acting beta-agonist; LABA: long-acting beta-agonist; SAMA: short-acting muscarinic antagonist; and LAMA: long-acting muscarinic antagonist.

**Table 3:** Percentages of success, error, and non-response per item of the CCTI questionnaire version 2.0 (n = 262).

Statements of the Questions of the CCTI Questionnaire	% Success *	% Error **	% Do Not Know/No Answer
1. The disease is treated by using inhalers daily	85.5	11.1	3.4
2. The effect of inhalers is to make the mucus more liquid	32.4	39.7	27.9
3. For the inhaler to be effective, I must feel that it enters my bronchial tubes.	10.7	78.2	10.7
4. The inhaler should only be used if I am breathlessness and I have a cold	71.4	24.4	3.8
5. It is better not to use the inhaler every day	67.2	22.9	9.5
6. Inhalers are used to open the bronchial tubes and let in more air	90.8	2.7	5.7
7. If I use the inhaler every day, I will get used to it and it will not work	68.7	13	18.3
8. The inhaler should be used as little as possible	64.5	29	6.5
9. When I get better, I have to stop using the inhaler	71.8	22.1	5.3
10. If I use inhalers daily, breathlessness will decrease	80.2	12.6	7.3

* Success considers a true statement true, and a false statement false; ** error considers a true statement false, or a false statement true.

**Table 4:** Analysis of the internal consistency of the CCTI questionnaire version 2.0.

Statements of the Questions of the CCTI Questionnaire	Mean of Scale If Item Is Removed	Scale Variance If Item Is Removed	Adjusted Item-Total Correlation	Cronbach’s Alpha If Element Is Removed
1. Inhalers taken daily are the treatment of the disease	7.50	5.87	0.29	0.59
2. The effect of inhalers is to make the mucus more liquid	7.54	4.98	0.24	0.61
3. For the inhaler to take effect, I must feel that it enters my bronchial tubes	8.10	5.29	0.26	0.59
4. The inhaler should only be used if I am breathlessness and I have a cold	7.63	5.51	0.33	0.57
5. It is better not to use the inhaler every day	7.56	5.40	0.32	0.57
6. Inhalers are used to open the bronchial tubes and let in more air	7.39	6.18	0.18	0.57
7. If I use the inhaler every day, I will get used to it and it will not work	7.37	5.38	0.32	0.57
8. The inhaler should be used as little as possible	7.65	5.35	0.34	0.57
9. When I get better, I have to stop using the inhaler	7.59	5.25	0.45	0.55
10. If I use inhalers daily, breathlessness will decrease	7.47	6.01	0.15	0.61

**Table 5:** Factor analysis of the CCTI questionnaire version 2.0.

Statements of the Questions of the CCTI Questionnaire	Factor 1: Beliefs Regarding Inhaler Use	Factor 2: Beliefs Regarding the Effect of Inhalers	Factor 3: Beliefs Regarding the Goal of Treatments
1. Inhalers taken daily are the treatment of the disease			0.58
2. The effect of inhalers is to make the mucus more liquid		0.56	
3. For the inhaler to take effect, I must feel that it enters my bronchial tubes		0.64	
4. The inhaler should only be used if I am breathlessness and I have a cold	0.68		
5. It is better not to use the inhaler every day	0.58		
6. Inhalers are used to open the bronchial tubes and let in more air		0.70	
7. If I use the inhaler every day, I will get used to it and it will not work	0.48		
8. The inhaler should be used as little as possible	0.66		
9. When I get better, I have to stop using the inhaler	0.74		
10. If I use inhalers daily, breathlessness will decrease			0.65

Varimax-rotated component matrix, numbers represent factor loading. Factor 1 (in light red) explained 21.76% of the total variance; factor 2 (in light blue) explained 12.18% of the total variance; and factor 3 (in light green) explained 12.64% of the total variance.

## Data Availability

The authors confirm that the data supporting the findings of this study are available within the article. The raw data can be requested for verification from the authors.
